# Supplementary material for: Pyruvate Dehydrogenase Kinase 1 inhibition mediated oxidative phosphorylation enhancement in cartilage promotes osteoarthritis progression
Source: BMC Musculoskelet Disord. 2023 Jul 20;24:597. doi: 10.1186/s12891-023-06585-6 (PMC10357736; doi:10.1186/s12891-023-06585-6)
Supplement: Supplementary file 1 — Additional file 1. [file 12891_2023_6585_MOESM1_ESM.zip › BP.Gsea.1653623667859/GOBP_PEPTIDYL_ASPARAGINE_MODIFICATION.html]

Details for gene set GOBP\_PEPTIDYL\_ASPARAGINE\_MODIFICATION[GSEA]

|  || Dataset | 55457\_collapsed\_to\_symbols.cls.cls #OA\_versus\_normal.cls.cls #OA\_versus\_normal\_repos |
| Phenotype | cls.cls#OA\_versus\_normal\_repos |
| Upregulated in class | OA |
| GeneSet | GOBP\_PEPTIDYL\_ASPARAGINE\_MODIFICATION |
| Enrichment Score (ES) | 0.59588593 |
| Normalized Enrichment Score (NES) | 1.8779833 |
| Nominal p-value | 0.004576659 |
| FDR q-value | 0.045643125 |
| FWER p-Value | 0.825 |
Table: GSEA Results Summary

  

Fig 1: Enrichment plot: GOBP\_PEPTIDYL\_ASPARAGINE\_MODIFICATION      
 Profile of the Running ES Score & Positions of GeneSet Members on the Rank Ordered List

  

| SYMBOL | TITLE | RANK IN GENE LIST | RANK METRIC SCORE | RUNNING ES | CORE ENRICHMENT || 1 | DDOST | dolichyl-diphosphooligosaccharide--protein glycosyltransferase non-catalytic subunit [Source:HGNC Symbol;Acc:HGNC:2728] | 428 | 0.695 | 0.1027 | Yes |
| 2 | DAD1 | defender against cell death 1 [Source:HGNC Symbol;Acc:HGNC:2664] | 737 | 0.585 | 0.1930 | Yes |
| 3 | ALG8 | "ALG8 alpha-1,3-glucosyltransferase [Source:HGNC Symbol;Acc:HGNC:23161]" | 900 | 0.543 | 0.2866 | Yes |
| 4 | ALG5 | ALG5 dolichyl-phosphate beta-glucosyltransferase [Source:HGNC Symbol;Acc:HGNC:20266] | 945 | 0.529 | 0.3866 | Yes |
| 5 | RPN2 | ribophorin II [Source:HGNC Symbol;Acc:HGNC:10382] | 1294 | 0.459 | 0.4494 | Yes |
| 6 | UBE2J1 | ubiquitin conjugating enzyme E2 J1 [Source:HGNC Symbol;Acc:HGNC:17598] | 1433 | 0.438 | 0.5243 | Yes |
| 7 | FUT8 | fucosyltransferase 8 [Source:HGNC Symbol;Acc:HGNC:4019] | 1565 | 0.418 | 0.5959 | Yes |
| 8 | ST6GAL1 | "ST6 beta-galactoside alpha-2,6-sialyltransferase 1 [Source:HGNC Symbol;Acc:HGNC:10860]" | 3502 | 0.202 | 0.4851 | No |
| 9 | MAGT1 | magnesium transporter 1 [Source:HGNC Symbol;Acc:HGNC:28880] | 3920 | 0.163 | 0.4845 | No |
| 10 | TUSC3 | tumor suppressor candidate 3 [Source:HGNC Symbol;Acc:HGNC:30242] | 3940 | 0.161 | 0.5146 | No |
| 11 | STT3A | STT3 oligosaccharyltransferase complex catalytic subunit A [Source:HGNC Symbol;Acc:HGNC:6172] | 4618 | 0.103 | 0.4823 | No |
| 12 | MGAT5 | "alpha-1,6-mannosylglycoprotein 6-beta-N-acetylglucosaminyltransferase [Source:HGNC Symbol;Acc:HGNC:7049]" | 5086 | 0.066 | 0.4588 | No |
| 13 | MGAT2 | "alpha-1,6-mannosyl-glycoprotein 2-beta-N-acetylglucosaminyltransferase [Source:HGNC Symbol;Acc:HGNC:7045]" | 5317 | 0.046 | 0.4500 | No |
| 14 | MGAT1 | "alpha-1,3-mannosyl-glycoprotein 2-beta-N-acetylglucosaminyltransferase [Source:HGNC Symbol;Acc:HGNC:7044]" | 5407 | 0.040 | 0.4508 | No |
| 15 | RPN1 | ribophorin I [Source:HGNC Symbol;Acc:HGNC:10381] | 6618 | -0.051 | 0.3669 | No |
| 16 | HIF1AN | hypoxia inducible factor 1 subunit alpha inhibitor [Source:HGNC Symbol;Acc:HGNC:17113] | 7815 | -0.142 | 0.3019 | No |
| 17 | UGGT2 | UDP-glucose glycoprotein glucosyltransferase 2 [Source:HGNC Symbol;Acc:HGNC:15664] | 8568 | -0.199 | 0.2824 | No |
| 18 | UGGT1 | UDP-glucose glycoprotein glucosyltransferase 1 [Source:HGNC Symbol;Acc:HGNC:15663] | 9516 | -0.275 | 0.2627 | No |
Table: GSEA details [plain text format]

  

Fig 2: GOBP\_PEPTIDYL\_ASPARAGINE\_MODIFICATION      
 Blue-Pink O' Gram in the Space of the Analyzed GeneSet

  

Fig 3: GOBP\_PEPTIDYL\_ASPARAGINE\_MODIFICATION: Random ES distribution      
 Gene set null distribution of ES for **GOBP\_PEPTIDYL\_ASPARAGINE\_MODIFICATION**
